# Accelerometer-Based Human Activity Recognition for Patient Monitoring Using a Deep Neural Network

**DOI:** 10.3390/s20226424

**Published:** 2020-11-10

**Authors:** Esther Fridriksdottir, Alberto G. Bonomi

**Affiliations:** Department of Patient Care & Measurements, Philips Research Laboratories, 5656AE Eindhoven, The Netherlands; efridriksdottir@ossur.com

**Keywords:** deep learning, human activity recognition (HAR), multiclass classification, patient monitoring, wearable sensors

## Abstract

The objective of this study was to investigate the accuracy of a Deep Neural Network (DNN) in recognizing activities typical for hospitalized patients. A data collection study was conducted with 20 healthy volunteers (10 males and 10 females, age = 43 ± 13 years) in a simulated hospital environment. A single triaxial accelerometer mounted on the trunk was used to measure body movement and recognize six activity types: lying in bed, upright posture, walking, wheelchair transport, stair ascent and stair descent. A DNN consisting of a three-layer convolutional neural network followed by a long short-term memory layer was developed for this classification problem. Additionally, features were extracted from the accelerometer data to train a support vector machine (SVM) classifier for comparison. The DNN reached 94.52% overall accuracy on the holdout dataset compared to 83.35% of the SVM classifier. In conclusion, a DNN is capable of recognizing types of physical activity in simulated hospital conditions using data captured by a single tri-axial accelerometer. The method described may be used for continuous monitoring of patient activities during hospitalization to provide additional insights into the recovery process.

## 1. Introduction

Hospitalized patients spend most of their time inactive and lying in bed [1,2,3]. This is especially concerning for older patients as physical inactivity following hospitalization can lead to functional decline [4]. On the other hand, stable or improved activity levels can serve as a valuable input for assessing patient discharge readiness [5]. Currently, monitoring mobility of hospitalized patients relies largely on direct observation from the caregivers. There are multiple tools available to assess the mobility and functional ability of patients. The choice of which assessment tool to use depends on feasibility and the clinician’s preference. These tools are mainly divided into two categories; self-report and performance-based measures [6]. Self-report questionnaires are easy to use and rapid, which makes them more preferable to performance-based measures [6]. However, self-report is based on the patient’s perception of their mobility rather than actual performance, which can lead to misleading results due to recall bias and under-reporting [7]. On the other hand, performance-based measures, such as timed “up and go” [8] or 6-minute walk test (6-MWT) [9], provide objective evidence about the capabilities of the patient. The downside of using performance-based measures is that setting up a test course requires equipment and measurements that can be time consuming for the clinician.

Wearable accelerometers have the potential to act as powerful tools in evaluating the health status of patients during recovery in an objective way and enabling evaluation of rehabilitation and other medical interventions [10]. Metrics such as amount of time spent in an upright position and daily step count have been found to have a relationship with length of hospital stay [5,11,12,13]. In addition, posture detection algorithms can provide important information for preventing pressure ulcer formation [14]. These metrics can be determined by using human activity recognition (HAR) based on camera systems or wearable sensors such as accelerometers, gyroscopes, magnetometers and barometric pressure sensors. Processing signals from wearable sensors requires considerably less computational power compared to the camera-based approach and imposes a less invasion of privacy. HAR using accelerometers embedded in smartwatches and smartphones as fitness trackers has recently become widely accepted in the consumer industry. However, step detection shows high error rates during slow walking and when using walking aid [15,16], which remains as one of the challenges for applying this technology in clinical settings, such as for patient monitoring.

HAR can be achieved by extracting hand-crafted features from sensor data and training classifiers that learn patterns and relationships between features and class labels. This is the traditional approach of using feature-based machine learning methods. Another approach that has become a popular choice for HAR recently is deep neural networks (DNNs). DNNs have developed and advanced considerably in recent years and has brought about breakthroughs in fields such as visual object recognition and natural language processing [17]. The advantage of using DNNs over conventional machine learning approaches is that they are able to automatically extract high-level features from raw input so hand-crafted feature extraction is not required.

This paper introduces a classification model that can recognize typical activities of patients during hospitalization using a single accelerometer mounted on the trunk. Two different approaches will be explored and compared; a deep learning approach and a feature-based machine learning approach. The aim is to investigate how accurately a deep learning algorithm can recognize activities typical for hospitalized patients using a single trunk-worn accelerometer.

## 2. Related Work

### 2.1. Methods Used for Human Activity Recognition (HAR)

#### 2.1.1. Feature-Based Approaches

Several acceleration features have been found to be valuable for HAR. These features are often based on the frequency of the signal or the statistical distribution of signal values.

A few examples are: tilt angle estimates to discriminate between lying and upright [10], discrete wavelet transform or vertical velocity estimates to recognize sit-to-stand transitions [18,19] and signal power to distinguish static activities from dynamic [10,20,21,22]. Machine learning classifiers, such as random forests, k-nearest neighbors and support vector machines, are often used to process acceleration features and classify activity types [23,24,25,26,27,28,29,30].

#### 2.1.2. Deep Neural Networks (DNNs)

Two types of DNN structures have been shown to perform well in accelerometer-based HAR in literature. These are convolutional neural networks (CNNs), a type of recurrent neural networks called long short-term memory (LSTM) and a combination of both. CNNs have been applied to sensor data for HAR with outstanding performances [31,32,33,34,35,36,37,38,39,40,41]. Previous studies proposed augmenting the feature vector extracted by a CNN with several statistical features [33,34]. Aviléz-Cruz et al. [35] developed a three-headed CNN model for recognizing six activities. The three CNNs work in parallel, all receiving the same input signal coming from a triaxial accelerometer and a triaxial gyroscope. The feature maps of the three CNNs are flattened and concatenated before they are passed into a fully connected layer and at last an output layer with a softmax activation.

Other studies have shown the relevance of using LSTM networks for HAR [36,42,43,44,45]. Lastly, a few studies have suggested augmenting CNNs with LSTM layers [37,46,47]. Karim et al. [37] proposed a model architecture in which a three-layer CNN and an LSTM layer extract features from sensor data in parallel. The resulting feature vectors are then concatenated and passed into a softmax classification layer. Others added LSTM layers after the CNN [46,47].

### 2.2. Human Activity Recognition (HAR) for Patient Monitoring

Using accelerometers for monitoring mobility of patients has been shown to be suitable for application in a clinical setting [10]. Aminian et al. [20] presented a rule-based HAR model comprising two accelerometers worn on the chest and thigh to classify lying, sitting, standing and dynamic activities. The model was tested on three hospitalized patients and compared to patient self-report. The authors found a significant discrepancy between the sensor outcome and patient self-report, which was explained by subjective bias of patients. The authors suggested that the thresholds used for classification should be adapted to each patient for improved performance. Rauen et al. [22] used a rule-based HAR model to monitor position changes of 30 immobile patients in early neuro-rehabilitation using triaxial accelerometers worn on the chest and thigh. The chest-worn accelerometer performed considerably better than the thigh sensor and was able to detect all position changes of the patients, and a few in addition to what was recorded in the standard written care documentation. The authors concluded that their approach was promising for monitoring position changes of immobile patients and evaluating their overall health.

## 3. Methods

### 3.1. Data Collection

Twenty healthy subjects, ten males and ten females (age =43±13 years, weight =78±15 kg, BMI =26±3 kg/m^2^) were recruited for the data collection. Inclusion criteria for volunteers was age in range of 18–65 years. This age range was selected to represent the typical age of hospitalized patients and a wide range of BMI were allowed for the participants in the study. Exclusion criteria were pregnancy, movement disorders, hypersensitivity to stainless steel and allergy to medical grade adhesives. The study, according to the regulations in the Netherlands, was waived as non-medical research and therefore approval by a IRB institution was not needed. The Internal Committee for Biomedical Experiments at Philips approved the study. Informed consent was obtained from all volunteers.

A GENEActiv (Activinsights Ltd., Kimbolton, UK) sensor was attached to the left side of the trunk of subjects by using a medical grade double adhesive. Careful orientation of the device allowed alignment of the *y*-axis of the GENEActiv device to the caudo-cranial direction of the body, resulting in alignment of the *x*-axis and the *z*-axis along the medio-lateral and antero-posterior direction of the body, respectively. This accelerometer placement was used as it proved to be an effective location for accelerometer-derived vital signs monitoring in patients. The sensor measured acceleration at 100 Hz sampling frequency with 12 bit resolution in the range of ±8 g (1 g = 9.8 m/s^2^). More wearable sensors were used to collect additional data during the measurement sessions, however, this data was not used for the classification models described in this paper. All sessions were recorded with a video camera for activity class label annotation purposes. Prior to the start of the data collection protocol, the accelerometers were all calibrated by orienting the sensor axis along the vertical direction to set the average signal to 1 g.

The protocol consisted of various activities typical for hospitalized patients such as lying in bed, eating and drinking, performing physiotherapy exercises and walking with and without walking aids at very slow to normal pace. A summary of the protocol can be found in Table 1 in chronological order. The order of activities was not randomized between subjects. The subjects were asked to act as a patient in the hospital (i.e., move slowly) for all tasks except for the Ebbeling test and the 6-MWT. That is because these tests were used to determine the subjects’ fitness and physical performance.

### 3.2. Data Preprocessing

Out of the 20 volunteers, one subject was not able to complete the 6-MWT and the walking up/down stairs activities due to fatigue. For another subject the acceleration signals during the 6-MWT had notably larger peaks than for all other subjects. That was due to one of the other devices used for data collection colliding with the GENEActiv sensor during this activity. The 6-MWT acceleration data for this subject was removed from the dataset because this periodic collision between devices is not expected during measurements outside the laboratory environment.

Activities were manually annotated and synced with the acceleration signal. Camera recordings were used to properly review volunteers activities during the protocol and generate annotations of start time and stop time for the various tasks. A single researcher reviewed the captured videos to generate activity label timestamps. The activities of the protocol were categorized into six activity classes; Lying, upright (sitting or standing), walking, stair ascent, stair descent and wheelchair transport. The dataset was split randomly into training, validation and test subsets based on participant IDs. Data from 50% of the subjects was used for training, 25% of the subjects for validation and 25% of the subjects for final testing.

Fixed-length sliding window technique, with length set to 6 seconds and 50% overlap, was used to segment the data. This segment length was chosen to make sure that relevant information was captured in each data segment during activities like slow walking and wheelchair transport. Indeed, for slow walking activities intervals of 6 seconds guaranteed the presence of at least 2 steps as well as for slow wheelchair activities movement were often repetitive on a 3–4 s period. Labels were assigned to each segment determined by class majority. Segments containing only unlabelled data or a majority of unlabelled data, such as during breaks between activities, were not used for training the classifiers.

### 3.3. Classification

**Figure 1:**
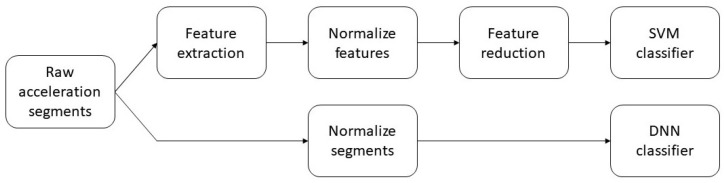
Flowchart showing the difference between handling the acceleration segments when using a feature-based machine learning classifier, in this case a support vector machine (SVM), and a deep neural network (DNN).

Two different classifiers were trained and their performances compared. The first classifier was a DNN that achieves automatic feature extraction from the normalized acceleration segments. The second classifier was a support vector machine (SVM) that required handcrafted features as input. Figure 1 shows the different data preparation needed for the two classification models.

#### 3.3.1. Deep Neural Network

**Figure 2:**
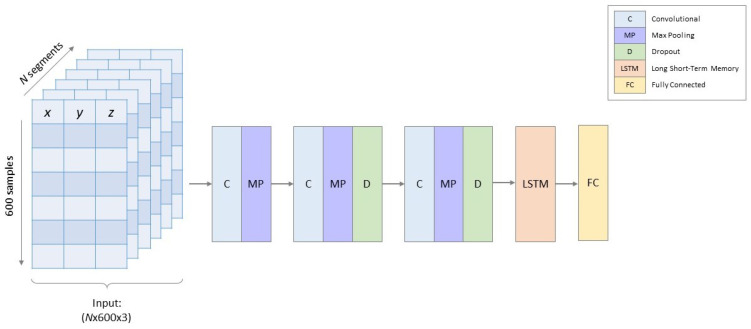
Model architecture of the deep neural network. Acceleration segments with dimension N×600×3, where *N* represents number of segments, were used as input. Batch normalization layers are not shown for simplicity. The dimensions of the feature maps before each feature extraction layer are noted below the layers.

Figure 2 shows the model architecture of the DNN. Normalized acceleration segments with dimensions 600×3 (6 s of *x*-, *y*- and *z*-acceleration sampled at 100 Hz) were used as input for the DNN. Three convolutional layers (filters: 8, 8 and 16 with kernel sizes: 23, 10 and 7, respectively) followed by an LSTM layer (units: 6) performed automatic feature extraction for the classification. The convolutional layers used a ReLu activation function and zero padding to avoid losing information at the boundaries of the input data. Max pooling layers (pool sizes: 10, 4 and 2, respectively), also with zero padding, and dropout layers (ratio: 30%) followed the convolutional layers to reduce risk of overfitting. Batch normalization layers were added after each convolutional layer as they have been shown to be effective in accelerating training of DNNs [45,48]. The last layer is a fully connected layer with a softmax activation that returns the classification predictions. The model was trained using an Adam optimizer [49] and batch size of 100. Hyperparameters such as number of filters, kernel size, pool size, dropout ratio and batch size was determined by iterating one hyperparameter at a time. The model was developed using Keras with TensorFlow backend.

Due to class imbalance, models were trained using a balanced batch generator the by imbalanced-learn library [50]. The purpose of the balanced batch generator was to make sure that in every batch there were equal amounts of samples from each class. The batch generator did so by creating copies of randomly selected samples belonging to all classes except the majority class of the batch.

#### 3.3.2. Feature-Based Classifier

A total of 86 features, from both time and frequency domains, were extracted from each acceleration segment. The features are listed in Table 2 and have previously been proposed for HAR [23,51,52]. Each feature was computed from the *x*-, *y*-, *z*-acceleration and the acceleration magnitude. Features were normalized to zero mean and unit standard deviation.

Prinicpal component analysis has commonly been used for reducing dimensionality of a feature set used for HAR [53,54,55,56]. By using the first 30 principal components, 99% of the cumulative variance of the original data can be maintained. A radial basis kernel was used and the γ parameter was set to γ=0.001. Class weights were inversely proportional to class size to deal with class imbalance. The classifier was implemented using Sklearn [57]. Feature normalization and development of the PCA transform parameters were obtained on the training dataset and then applied to the validation and testing datasets.

## 4. Results

The dataset contained approximately 23,000 labelled segments in total. Roughly 64% of the segments belonged to the walking class while the wheelchair class, which was smallest class, accounted for less than 2% of all the samples. Both the DNN and SVM classifiers were evaluated on the same holdout dataset containing data from 25% of the subjects. Table 3 shows the performance scores of both classification models. The DNN reached a considerably better performance with 94.5% in overall accuracy compared to 83.35% for the SVM. The between-subject variability in the DNN classification accuracy within the holdout dataset was 6%. F1-score is often considered a better metric when dealing with classification problems of imbalanced datasets and is therefore listed in the table.

**Figure 3:**
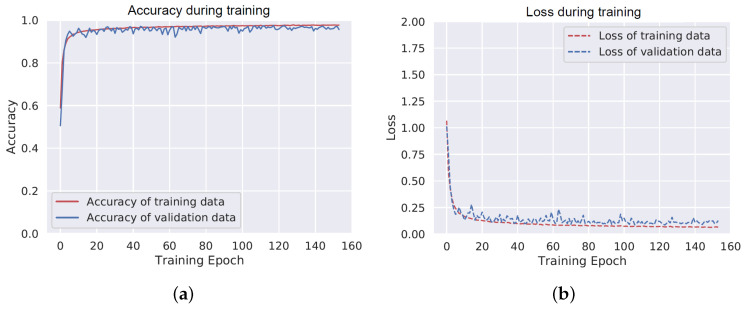
Learning curves during training of the deep neural network (DNN). (**a**) Accuracy of the training and validation data, (**b**) loss of the training and validation data.

Figure 3 shows the accuracy and loss of the DNN model on training and validation datasets. The performance of the model stops improving around the 50th training epoch. The model performs similarly for the training data and the validation data, which indicates low risk of overfitting.

**Figure 4:**
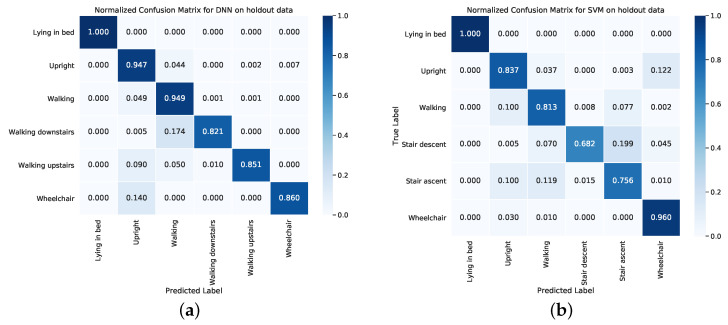
Normalized confusion matrices on holdout data of (**a**) the deep neural network (DNN) and (**b**) the support vector machine (SVM) classifier.

Figure 4a shows the confusion matrix resulting from applying the DNN to the holdout data. Lying in bed was correctly classified for 100% of the segments. Segments labelled as upright and walking were correctly classified 94.7% and 94.9% of the time, respectively. The stair ascent, stair descent and wheelchair classes had slightly poorer classification rates of 82.1%, 85.1% and 86%, respectively. For comparison, the confusion matrix of the SVM on the same holdout data is shown in Figure 4b. The classification rate of the SVM is less for all classes except for lying in bed and wheelchair.

**Figure 5:**
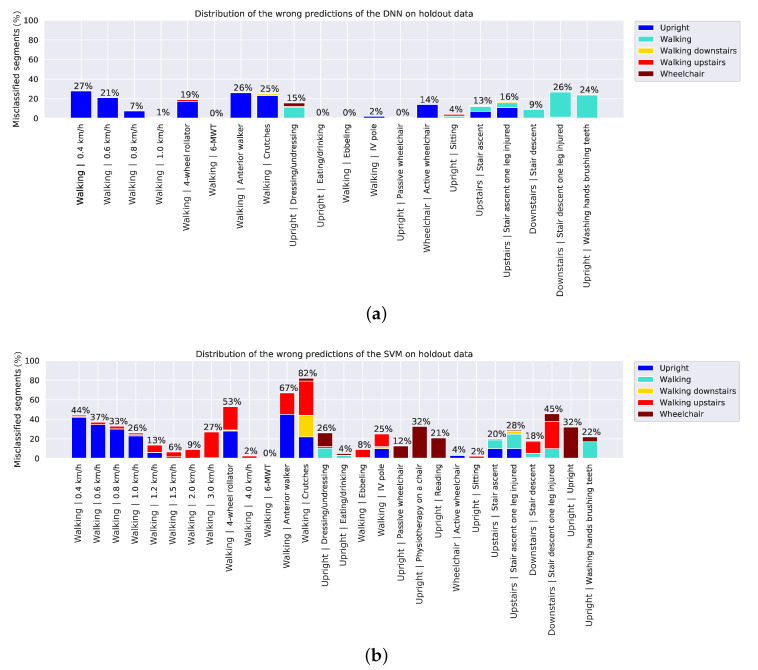
Percentage of wrong predictions per activity by (**a**) the deep neural network (DNN) and (**b**) the support vector machine (SVM). The colors represent the wrongly predicted class.

Figure 5 shows the percentage of wrongly classified segments per activity of the holdout dataset to indicate which activities are more difficult to classify than others. Slow walking, walking with walking aid and walking up/down stairs are the most challenging activities to classify for both models.

## 5. Discussion

This study demonstrated that a DNN model could be used to accurately classify activities that are typical for hospitalized patients using an accelerometer worn on the trunk. The DNN model showed substantially larger accuracy than a feature-based SVM on the presented laboratory data. Continuous patient monitoring using this approach could add insight into the recovery process by providing objective information about patients’ mobility and behavior. The DNN architecture was relatively small with 3 convolutional layers, a recurrent layer and a final dense layer. This model architecture and the number of operations required for real time data processing make the implementation of the DNN feasible for embedded processing in wearable devices equipped with modern processors capable of running computing libraries such as TensorFlow Lite [58].

Monitoring patient activity requires accurate walking detection at slow speeds as patients often ambulate at less than 1 km/h [59]. At very slow walking speeds, both classifiers had difficulties detecting walking. The DNN misclassified 27% of segments in the holdout dataset representing walking at 0.4 km/h as upright position. The ratio of misclassified segments improved as speed increased and for speeds higher than 1 km/h, 100% of the segments were correctly classified as walking. Segments representing walking with a 4-wheel rollator, walker and crutches were misclassified as upright for 18% to 26% of the segments. Activities while standing such as dressing/undressing, washing hands and brushing teeth were sometimes mistaken as walking or wheelchair. That may be due to small movements that resemble acceleration signals belonging to those two classes. The walking up/down stairs activities had 9% to 26% misclassification rates, which was expected partly because the acceleration signals while walking up/down stairs resemble those during walking in the corridor. In addition, in between floors there were parts where the subjects had to walk a few steps on a flat level before continuing walking up/down the stairs. These short flat level parts were not specifically annotated and therefore it is possible that there were some segments labelled as walking up/down stairs that should have been labelled as walking.

The amount of misclassified segments is considerably higher for the SVM. Walking with crutches was the activity with the highest percentage of misclassifications, in total 82%. These segments where misclassified as upright, wheelchair, stair ascent and descent. Walking with an anterior walker and 4-wheel rollator follow with misclassification rates 67% and 53%, respectively. Many of the activities while standing or sitting, such as dressing, undressing, physiotherapy and reading, were falsely predicted as belonging to the wheelchair class. The difficulties of the SVM in predicting walking with walking aid and the wheelchair class might indicate that different features were needed for these patient-specific activities.

**Figure 6:**
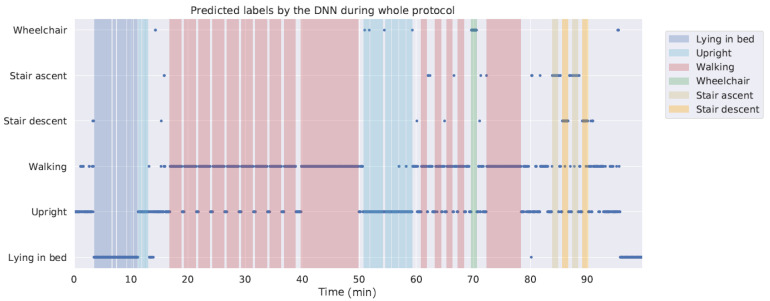
Predictions of the deep neural network (DNN) when the whole recording session of one subject is passed into the model. The grey areas represent unlabelled activities, which were not included when training the model.

A limitation of this study was that the algorithm was trained and tested using laboratory data. Previous studies have shown that performance of algorithms in laboratory conditions may not accurately reflect performance in daily life [60]. This especially applies to algorithms such as DNNs that require large and representative datasets for generalizing. However, preliminary testing including the unlabelled activities from the dataset collected for this study indicates good performance on new data, with just a few false positives for wheelchair and stair walking activities. Figure 6 shows the predictions of the DNN classifier for segments of the whole recording session of a representative participant from the holdout dataset. Another limitation is that this study does not address the challenge of monitoring changes in activity pattern in patients which is an important target when looking into clinical applicability of the presented model to support assessment of patient recovery during hospitalization.

## 6. Conclusions

This work showed that a single trunk-worn accelerometer has the potential to monitor mobility of patients in hospitals. The DNN model presented in this report is a reliable algorithm for recognizing activities that are typical of daily patient behavior in the hospital. The model can accurately detect walking at speeds down to 1 km/h. This method has the potential to provide nurses and doctors insight into the recovery process of their patients and valuable objective information for making decisions regarding patient discharge. Future studies are needed to validate the classification model in continuous monitoring of hospitalized patients.

## Figures and Tables

**Table 1:** Activities used in the study protocol, with corresponding class labels and duration per participant. 6-MWT stands for 6-minute walk test.

Activity Label	Class Label	Duration (mm:ss)
**Activities in and around bed**		
Lie supine	Lying in bed	3:00
Lie left	Lying in bed	0:30
Lie right	Lying in bed	0:30
Restless in bed	Lying in bed	1:00
Physiotherapy in bed	Lying in bed	1:00
Reclined	Lying in bed	0:30
Upright	Upright	0:30
Sitting edge of bed	Upright	0:30
Standing next to bed	Upright	0:30
**Treadmill activities**		
0.4 km/h	Walking	2:00
0.6 km/h	Walking	2:00
0.8 km/h	Walking	2:00
1.0 km/h	Walking	2:00
1.2 km/h	Walking	2:00
1.5 km/h	Walking	2:00
2.0 km/h	Walking	2:00
3.0 km/h	Walking	2:00
4.0 km/h	Walking	2:00
Ebbeling	Walking	∼10:00
**Activities of daily hospital living**		
Dressing/undressing	Upright	1:00
Reading	Upright	1:00
Physiotherapy on a chair	Upright	1:00
Eating/drinking	Upright	1:00
Sit-to-Stand transitions	Upright	1:00
**Hospital ambulation**		
Patient transport in wheelchair	Upright	1:00
Washing hands brushing teeth	Upright	1:00
Crutches	Walking	1:00
Anterior walker	Walking	1:00
IV pole	Walking	1:00
4-wheel rollator	Walking	1:00
Self propelled wheelchair	Wheelchair	1:00
6-MWT	Walking	6:00
**Stair walking**		
Stair ascent one leg injured	Stair ascent	1:00
Stair descent one leg injured	Stair descent	1:00
Stair ascent	Stair ascent	1:00
Stair descent	Stair descent	1:00

**Table 2:** The features extracted from each acceleration segment. Each feature was extracted from four signals; the *x*-, *y*-, *z*-acceleration and the acceleration magnitude.

Feature	Description
Mean	Mean value of the vector
Absolute mean	Mean of absolute values in the vector
Median	Median value of the vector
Mean absolute deviation	Mean absolute deviation of the vector
Standard deviation	Standard deviation of the vector
Variance	Variance of the vector
Minimum value	Lowest value in the vector
Maximum value	Highest value in the vector
Full range	Difference between the maximum and minimum value of the vector
Interquartile range	Difference between the 1st and 3rd quartile
Area	Sum of all values in the vector
Absolute area	Sum of all absolute values in the vector
Energy	Sum of squared components of the vector
Correlation	Correlation coefficients between each pair of vectors
Skewness	Shape of distribution
Kurtosis	Shape of distribution
Spectral entropy	A measure of the complexity of a signal
Spectral centroid	Mean of fourier transform
Spectral variance	Variance of fourier transform
Spectral skewness	Skewness of fourier transform
Spectral kurtosis	Kurtosis of fourier transform

**Table 3:** Classification performance of the deep neural network (DNN) and support vector machine (SVM) on holdout data. Precision, recall and F1-scores are reported as weighted averages.

	Accuracy	Precision	Recall	F1-Score
DNN	0.9452	0.9507	0.9452	0.9464
SVM	0.8335	0.8919	0.8335	0.8507
